# Identification and validation of potential genes for the diagnosis of sepsis by bioinformatics and 2-sample Mendelian randomization study

**DOI:** 10.1097/MD.0000000000038917

**Published:** 2024-07-19

**Authors:** Zhongbo Xu, Lin Li

**Affiliations:** a Emergency Department, Affiliated Hospital of Jiangxi University of Chinese Medicine, Nanchang, Jiangxi, China.

**Keywords:** machine learning, MCEMP1, Mendelian randomization, sepsis, weighted gene co-expression network analysis

## Abstract

This integrated study combines bioinformatics, machine learning, and Mendelian randomization (MR) to discover and validate molecular biomarkers for sepsis diagnosis. Methods include differential expression analysis, weighted gene co-expression network analysis (WGCNA) for identifying sepsis-related modules and hub genes, and functional enrichment analyses to explore the roles of hub genes. Machine learning algorithms identify 3 diagnostic genes - CD177, LDHA, and MCEMP1 - consistently highly expressed in sepsis patients. The nomogram model effectively predicts sepsis risk, supported by receiver operator characteristic (ROC) curves. Correlations between diagnostic genes and immune cell infiltration are observed. MR analysis reveals a positive causal relationship between MCEMP1 and sepsis risk. In conclusion, this study presents potential sepsis diagnostic biomarkers, highlighting the genetic association of MCEMP1 with sepsis for insights into early diagnosis.

## 1. Introduction

Sepsis is a severe infectious disease characterized by a systemic inflammatory response syndrome triggered by infection, leading to multiple organ dysfunction and inadequate tissue perfusion.^[1]^ The global incidence and mortality rates of sepsis have garnered significant attention in the medical field due to recent changes in global health conditions and the increase in antibiotic misuse.^[2]^ The early diagnosis of sepsis remains a clinical challenge. Standard physiological parameters for preliminary diagnosis include body temperature, heart rate, respiratory rate, and inflammatory markers such as C-reactive protein and procalcitonin.^[3]^ However, these indicators lack specificity, especially in the early stages of sepsis, where atypical symptoms in patients increase the risk of misdiagnosis and mistreatment. The absence of early definitive diagnostic markers often results in antibiotic selection relying on broad-spectrum antibiotics, potentially contributing to antibiotic misuse and increased antibiotic resistance. Recognizing the urgent need for more precise and sensitive biomarkers to support early diagnosis and treatment of sepsis, there is a pressing requirement to develop accurate and rapid diagnostic methods for sepsis.

The rapid development of bioinformatics and machine learning has provided a new perspective for addressing the diagnostic challenges of sepsis.^[4,5]^ Recent studies indicate that machine learning algorithms, through the integration of large-scale genomic data, can learn and identify gene expression patterns closely associated with sepsis.^[6,7]^ This discovery offers hope for uncovering potential biomarkers, thereby opening up new avenues for the early diagnosis of sepsis. In recent research, bioinformatics methods have been employed to investigate core genes related to sepsis occurrence, development, and diagnosis. These genes involve critical biological processes such as immune response, inflammation regulation, and cell signal transduction.^[8]^ Despite these advancements, it is essential to recognize that relying solely on the results of bioinformatics and machine learning analyses may not furnish sufficient evidence to confirm the causal relationship between the selected genes and sepsis.

Mendelian randomization (MR) has gained widespread recognition as a reliable method for inferring potential causal relationships.^[9]^ This methodology employs single nucleotide polymorphisms (SNPs) as instrumental variables (IVs) for evaluating the causal relationship between exposure factors and observed outcomes.^[10]^ Employing genetic variants strongly associated with exposure factors as instrumental variables, MR allows for the inference of causal effects between exposure factors and study outcomes. Studies have consistently shown that applying MR analysis in genetic epidemiological research yields more reliable causal evidence and effectively eliminates the influence of confounding factors, further enhancing the credibility of research conclusions.^[11]^

In this study, we utilized weighted gene co-expression network analysis (WGCNA) to identify the most relevant feature genes associated with sepsis. Subsequently, 3 machine learning methods were employed to screen 3 key genes (MCEMP1, CD177, and LDHA) as potential diagnostic markers. We evaluated the diagnostic efficacy through Receiver Operating Characteristic (ROC) curve analysis and nomogram construction. Finally, we investigated the causal relationship between MCEMP1 and sepsis using MR studies. By integrating these methodologies, we aim to offer innovative and reliable molecular markers for the early diagnosis of sepsis.

## 2. Materials and methods

Given that all data are from publicly accessible databases, our study does not require additional approval for independent ethical review.

### 2.1. Acquiring data

In the gene expression omnibus database at https://www.ncbi.nlm.nih.gov/gds/,^[12]^ search for “sepsis” and select “Homo sapiens” as the species. The search results identified 4 expression profile datasets that include whole blood samples from healthy individuals and patients with sepsis. The information about these distinct gene expression datasets is presented in Table 1. Specifically, GSE26378, GSE28750, and GSE64457 serve as training datasets, while GSE66099 is the validation set. The inclusion criteria for the datasets were as follows: microarray datasets from genome-wide gene expression profiles of blood, with the additional inclusion of microarray datasets from human sepsis samples and healthy state samples. The exclusion criteria were as follows: samples combined with other diseases and sample sizes of <10 in the sepsis and normal groups.

**Table 1:** Descriptive of the GEO datasets.

GEO accession	Platform	Samples (number)		Attribute
		Health	Sepsis	
GSE26378	GPL570	21	82	Training
GSE28750	GPL570	20	10	Training
GSE64457	GPL570	8	9	Training
GSE66099	GPL570	47	18	Validation

GEO = gene expression omnibus.

### 2.2. Differentially expressed genes analysis

The R software “SVA” package was employed to normalize the expression values of 3 datasets (GSE26378, GSE28750, and GSE64457) and merge the data.^[13]^ Principal component analysis (PCA) was then utilized to assess the elimination of batch effects. Subsequently, the “limma” package was applied for differential analysis of the datasets to identify differentially expressed genes (DEGs) between the healthy and sepsis groups.^[14]^ Screening conditions were set at |log FC|≥1 and corrected *P* < .05.

### 2.3. WGCNA

WGCNA is a frequently utilized modular analysis technique to identify and screen biomarkers or drug targets for complex diseases.^[15]^ It is also applied to identify highly covariant gene expression matrices, offering new disease insights. In this study, we employed the “WGCNA” package in the R language to analyze the co-expression network for the corrected genes.^[16]^ Genes exhibiting similar expression patterns were extracted based on various gene expression profiles. Subsequently, modules with the highest correlation to sepsis and all characteristic genes within these modules were identified.

### 2.4. Identification of hub genes and comprehensive functional enrichment evaluation

Combining DEGs with the module genes identified through WGCNA, we pinpointed hub genes closely linked to sepsis pathogenesis. The Venn diagram was constructed using the R software. Subsequently, we delved into these hub genes’ biological pathways and functions utilizing the “clusterProfiler” package in R.^[17]^ Initially, we scrutinized 3 categories of biological processes—Biological Process (BP), Molecular Function (MF), and Cellular Component (CC)—via Gene Ontology (GO) functional analysis. We then utilized the Kyoto Encyclopedia of Genes and Genomes (KEGG) to evaluate the biological pathways associated with the identified hub genes.

### 2.5. Screen diagnostic genes

Sepsis diagnostic genes were identified by screening with 3 machine learning algorithms: random forests (RF),^[18]^ least absolute shrinkage and selection operator (LASSO) logistic regression,^[19]^ and support vector machine-recursive feature elimination (SVM-RFE).^[20]^ Subsequently, the characteristic genes obtained from these methods were intersected to derive the diagnostic genes, and their patterns were visualized using R software.

### 2.6. Validation of diagnostic genes

To validate the reliability and repeatability of these diagnostic genes, we employed GSE66099. Using R software, we assessed the expression of characteristic genes in both the training and validation sets. Subsequently, we evaluated diagnostic accuracy by constructing ROC curves. The area under the curve (AUC) was calculated, with a larger AUC indicating superior predictive performance within the range of 0 to 1.

### 2.7. Construction of a nomogram

We will generate a nomogram using the R software “rms” package to facilitate the derivation of disease diagnosis probability using diagnostic genes. A nomogram is a quantitative analysis chart illustrating the functional relationship between multiple variables through disjoint line segments in plane coordinates.^[21]^ These line segments are drawn based on specific proportions, enabling the convenient calculation of an individual risk probability. We evaluated the nomogram performance and internal effectiveness by creating a calibration curve and conducting a decision curve analysis.

### 2.8. Immune infiltration assessment

Gene expression profiles of 22 immune cell types were acquired from the CIBERSORT database (https://cibersort.stanford.edu/) by implementing R software.^[22]^ The proportional representation of these immune cells in 2 distinct groups was computed using the “e1071” package in R. Subsequently, these proportions were visualized in a plot to illustrate the extent of immune cell infiltration. To elucidate the correlation between the distribution of immune cell infiltration and the disparities observed, a correlation heatmap and a violin plot were generated employing the “corrplot” and “vioplot” packages, respectively. A *P* value threshold of <.05 was set to determine statistically significant differences between the groups.

### 2.9. MR analysis

This study exclusively used data from publicly available genome-wide association study (GWAS) datasets, employing a 2-sample MR method with SNPs as instrumental variables to investigate the causal link between the diagnostic gene (MCEMP1) and sepsis. The MR analysis, conducted using the “TwoSampleMR” package, applied the inverse variance-weighted (IVW) method to assess the relationship between diagnostic gene levels and sepsis risk, with additional sensitivity analysis using the MR-Egger method.^[23]^ MR results were presented as odds ratios (OR) and 95% confidence intervals (95% CI), and results were considered statistically significant when *P* < .05.

## 3. Results

### 3.1. Identification of DEGs

**Figure 1.**
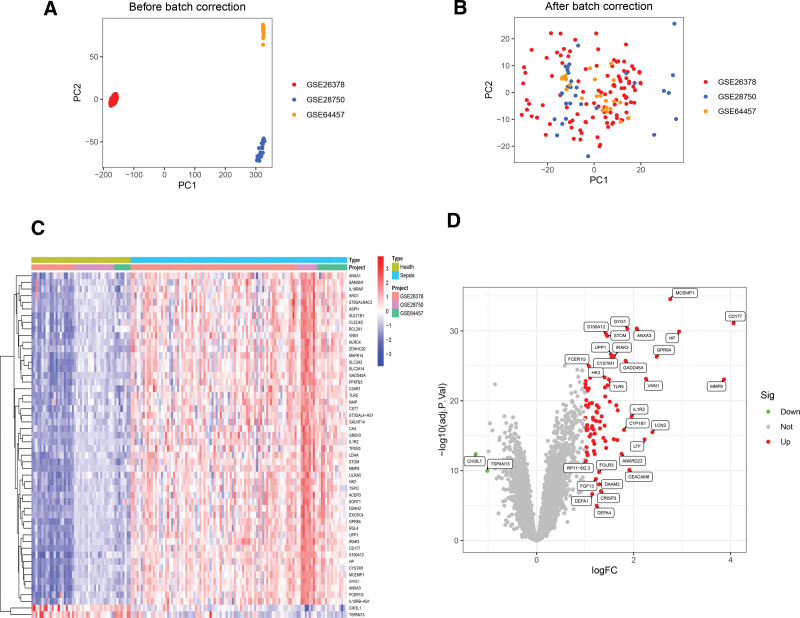
Batch normalize and DEGs expression levels of 3 datasets (GSE26378, GSE28750, and GSE64457). (A, B) PCA before and after batch correction. (C, D) Heat map and volcano plot of DEGs. DEGs = differentially expressed genes, PCA = principal component analysis.

The gene expression matrix was normalized by integrating the GSE26378, GSE28750, and GSE64457 datasets, eliminating batch differences. PCA clustering plots were generated before and after this process to visually assess the impact of batch normalization, as depicted in Figure 1A and B. After eliminating batch differences, the results demonstrate a more distinct clustering between the 2 datasets, indicating a substantial reduction in batch-related variations. 83 sepsis-related DEGs were identified, comprising 81 upregulated genes and 2 down-regulated genes. We then generated heat maps and volcano plots for visualization (Fig. 1C and D).

### 3.2. Screening for key modules by WGCNA

**Figure 2.**
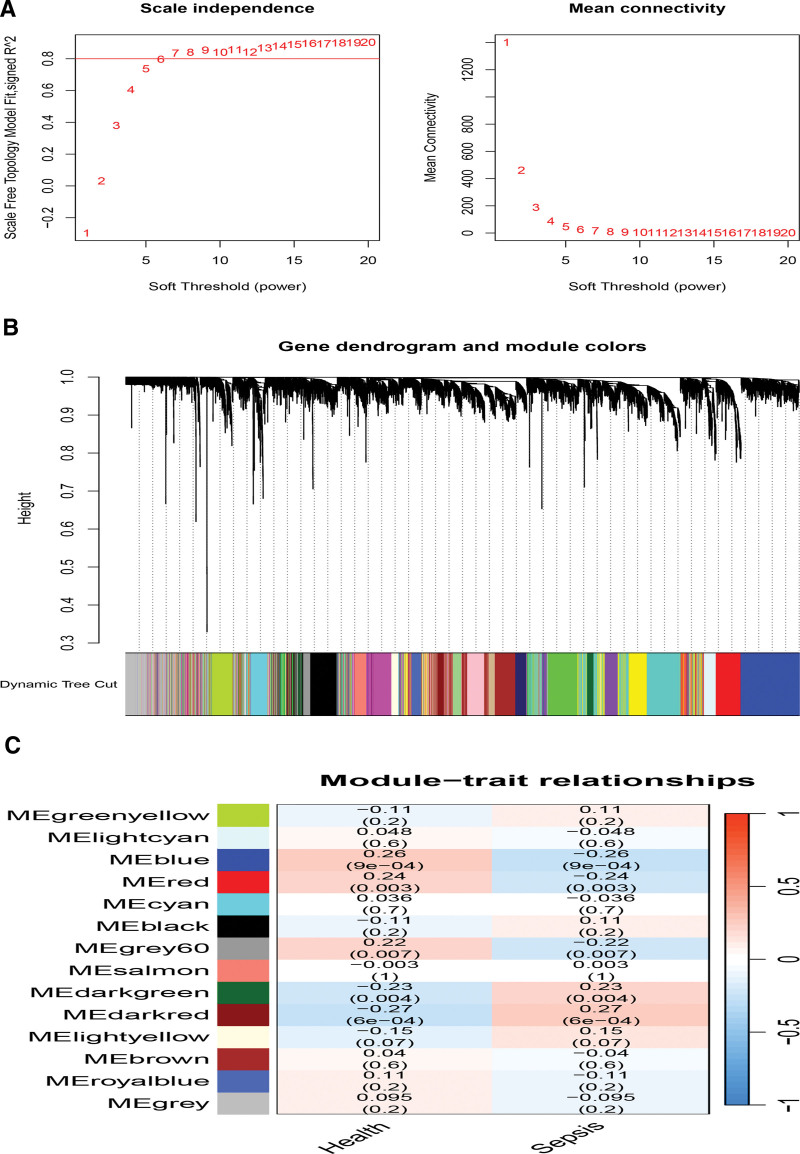
WGCNA of sepsis. (A) Determination of soft-threshold power for sepsis. (B) Cluster dendrogram of sepsis highly connected genes in key modules. (C) Relationships between modules and traits in sepsis. Correlations and *P* values are included in each cell. WGCNA = weighted gene co-expression network analysis.

Applying WGCNA, we calculated the parameter “power” within the 1 to 30 R² to achieve a scale-free network. A higher R² value indicates a better fit to the scale-free distribution, with a typical recommendation of R²≥ 0.8. The value first reached 0.8 when the power parameter was set to 6 (Fig. 2A). Subsequently, an adjacency matrix was generated using the adjacency function. Hierarchical clustering was performed using the topological overlap measure (TOM) dissimilarity (Fig. 2B). Fourteen co-expression modules were identified, and those with a significance level (*P* < .05) were considered vital modules. As shown in Figure 2C, the MEblue module exhibited the strongest positive correlation and comprised 1816 genes.

### 3.3. Analysis of the hub genes and functional enrichment

**Figure 3.**
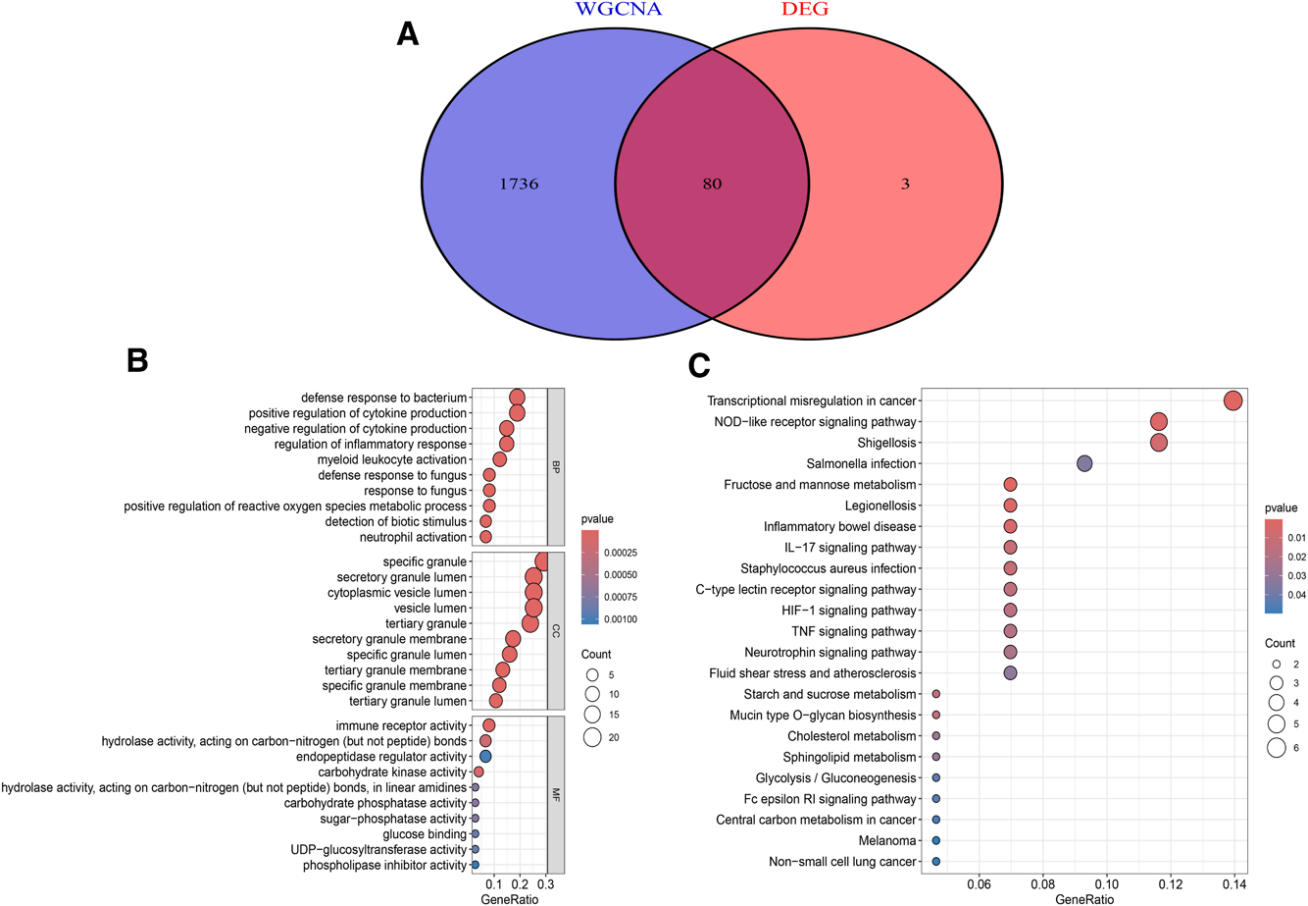
Shared hub genes and functional enrichment analysis. (A) Venn diagram of displaying 2 common genes between the WGCNA and DEGs. (B) Column diagrams of GO enrichment analysis. (C) Column diagrams of KEGG enrichment analysis. DEGs = differentially expressed genes, WGCNA = weighted gene co-expression network analysis.

The DEGs intersected with the gene ensemble housed within the MEblue module, identifying 80 hub genes (Fig. 3A). We conducted comprehensive Gene Ontology (GO) enrichment and Kyoto Encyclopedia of Genes and Genomes (KEGG) pathway analyses to enhance our comprehension of the biological processes and pathways intricately linked to these hub genes. The GO enrichment analysis highlighted the preeminent enrichments in “defence response to bacterium,” “specific granule,” and “immune receptor activity” (Fig. 3B). Concurrently, the KEGG pathway analysis underscored the prominence of pathways such as the “NOD − like receptor signaling pathway,” “IL − 17 signaling pathway,” and “C − type lectin receptor signaling pathway” (Fig. 3C).

### 3.4. Selection of diagnostic genes

**Figure 4.**
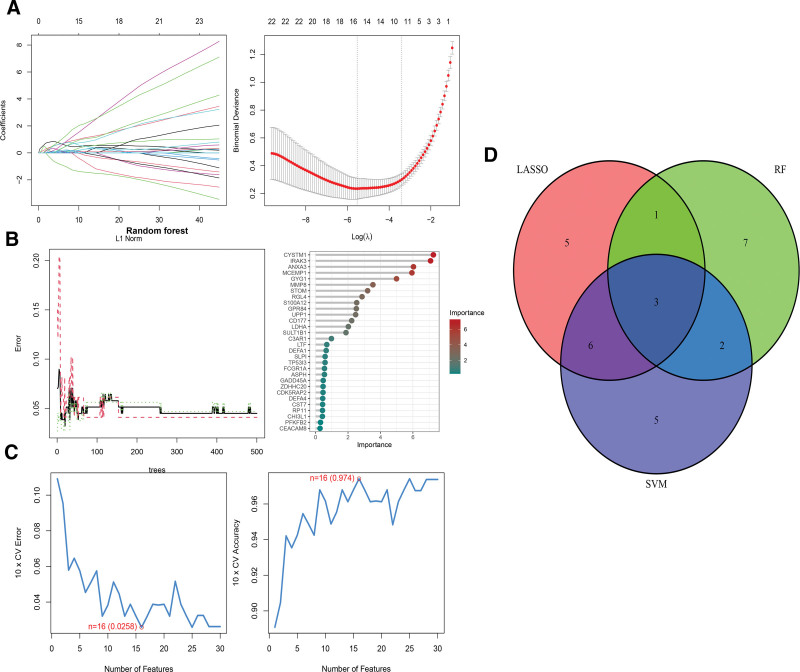
Screening for diagnostic genes using machine learning algorithms. (A) LASSO logistic regression algorithm to screen diagnostic markers; different colors represent different marker. (B) Relationship between the number of random forest trees and the error rate, and ranking of the relative importance of hub genes. (C) Based on support vector machine-recursive feature elimination (SVM-RFE) algorithm to screen biomarkers. (D) Venn diagram displaying 2 common genes from the LASSO, RF and SVM-RFE models. LASSO = least absolute shrinkage and selection operator, RF = random forests, SVM-RFE = support vector machine-recursive feature elimination.

The hub genes were further screened using LASSO regression, RF, and SVM-RFE algorithms. The LASSO regression model was constructed and cross-validated, revealing the minimum error value corresponding to 15 feature genes (Fig. 4A). Subsequently, a comprehensive analysis using the RF method on hub genes yielded 13 genes, identified based on an importance score exceeding 2 (Fig. 4B). Simultaneously, the SVM-RFE algorithm, employing 10-fold cross-validation, selected 16 feature genes (Fig. 4C). The 3 characteristic genes (MCEMP1, CD177, and LDHA) were obtained by intersecting the results from the 3 methods, as shown in Figure 4D. This integrated approach enhances the reliability of diagnostic gene selection.

### 3.5. Diagnostic gene expression and diagnostic capability validation

**Figure 5.**
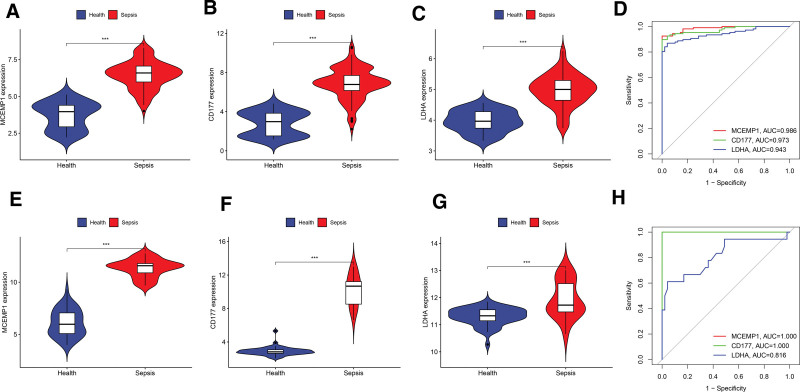
Validation analysis of diagnostic genes. (A-C) Violin plot of diagnostic genes expression in health and sepsis in the training set. *P < .05; **P < .01; ***P < .001. (E-G) Violin plot of diagnostic genes expression in health and sepsis in the validation set. *P < .05; **P < .01; ***P < .001. (D) ROC analysis of diagnostic genes in training set. (H) ROC analysis of diagnostic genes in validation set. ROC = receiver operator characteristic.

The expression differences of the 3 characteristic genes between the sepsis and healthy groups were evaluated in the training datasets. The results revealed a significant up-regulation of MCEMP1, CD177, and LDHA in the sepsis groups compared to the healthy groups (*P* < .05) (Fig. 5A–C). This trend in gene expression was consistently replicated in the independent validation dataset (Fig. 5E–G), fortifying the robustness and reliability of the results.

In addition, ROC curves illustrated that AUC values for the characteristic genes in the training dataset exceeded 0.9 (Fig. 5D). Furthermore, the AUC values for the signature genes in the validation dataset consistently surpassed 0.9, underscoring their robust predictive efficacy in distinguishing between sepsis and healthy groups (Fig. 5H).

### 3.6. Construction and evaluation of nomogram model

**Figure 6.**
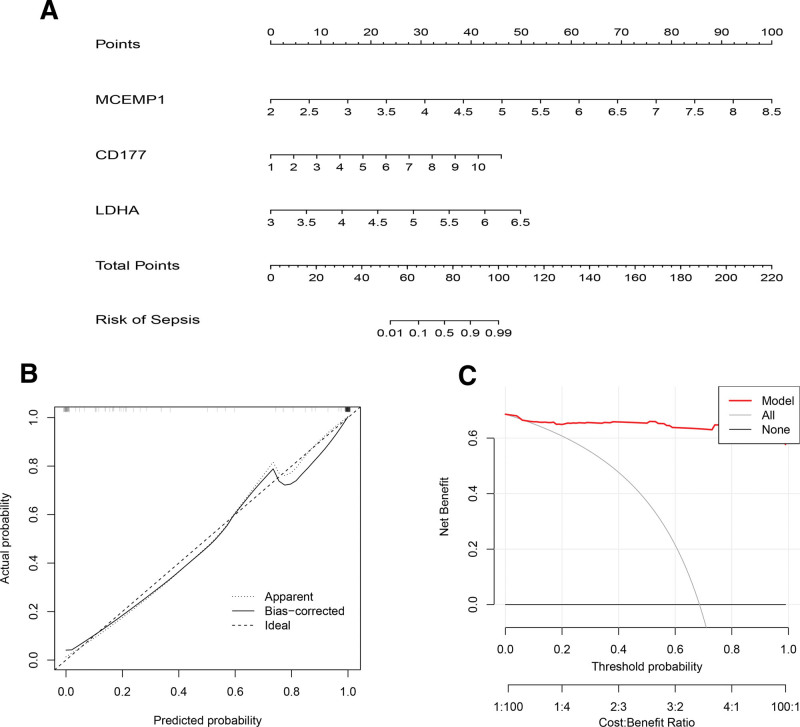
Construction and validation of a nomogram model (A) The nomogram of diagnostic genes to predict the occurrence of sepsis. (B) The calibration curve to assess the predictive power of the nomogram model. (C) The DCA curve to evaluate the clinical application value of nomogram model. DCA = decision curve analysis.

Logistic regression was employed to construct prediction models using 3 matrices of diagnostic gene expressions, visually represented through nomogram plots (Fig. 6A). The results of calibration curve analysis demonstrated the nomogram model accurate predictive ability (Fig. 6B). Additionally, decision curve analysis suggested that decisions informed by the nomogram model might offer potential benefits for sepsis patients (Fig. 6C).

### 3.7. Immune infiltrate analysis

**Figure 7.**
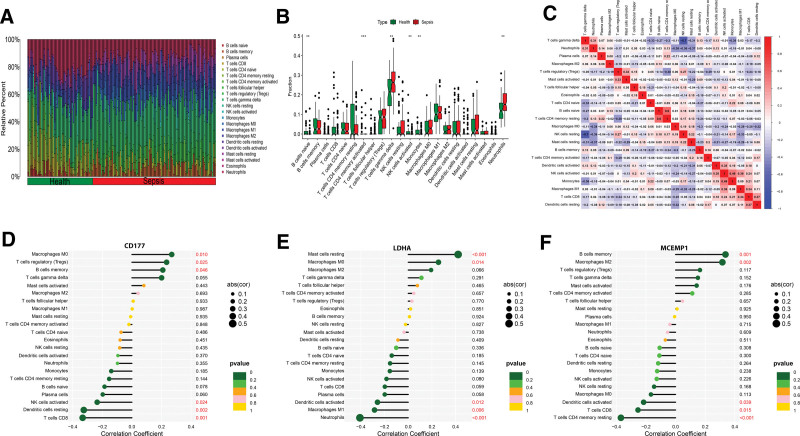
Results of immune infiltration by CIBERSORT. (A) Bar-plot showed the composition of 22 kinds of immune cells. (B) Violin diagram presented the difference of immune infiltration of 22 kinds of immune cells. (C) Correlation heatmap of 22 immune cell subpopulations. Red and blue represent positive and negative correlation, respectively. The rectangle with a deeper color has a stronger correlation index. (D-F) Correlation between immune infiltrating cells and diagnostic genes.

A matrix depicting the content of immune cells in both the health and sepsis groups was formulated, revealing variations in the distribution of infiltrating 22 immune cell types across a diverse set of samples, as illustrated in Figure 7A. Violin plots were generated to examine differences in the infiltrations of the 22 immune cell types between the 2 groups (Fig. 7B). Statistically significant disparities were identified in the infiltrations of “B cells naive,” “T cells CD4 memory activated,” “T cells gamma delta,” “NK cells activated,” “Monocytes,” and “Neutrophils.” “B cells naive,” “T cells gamma delta,” and “Neutrophils” were upregulated in sepsis samples. Conversely, “T cells CD4 memory activated,” “NK cells activated,” and “Monocytes” were down-regulated in sepsis samples. The immune cell correlation heatmap analysis disclosed a notably strong positive correlation between “B cells naive” and “T cells CD4 memory resting” (correlation coefficient *R* = 0.6). Conversely, the most pronounced negative correlation was observed between “NK cells resting” and “T cells gamma delta” (correlation coefficient r = −0.7), as depicted in Figure 7C.

In addition, Spearman correlation analysis between 3 diagnostic genes and 22 immune cells revealed that CD177 exhibited the strongest positive correlation with “Macrophages M0” and the most substantial negative correlation with “T cells CD8” (Fig. 7D). “Mast cells resting” and “Neutrophils” demonstrated the most pronounced positive and negative correlations with LDHA (Fig. 7E). The most significant correlations for MCEMP1 were observed with “B cells memory” and “T cells CD4 memory resting,” with positive and negative correlations, respectively (Fig. 7F).

### 3.8. MR analysis of the MCEMP1 and sepsis risk

**Figure 8.**
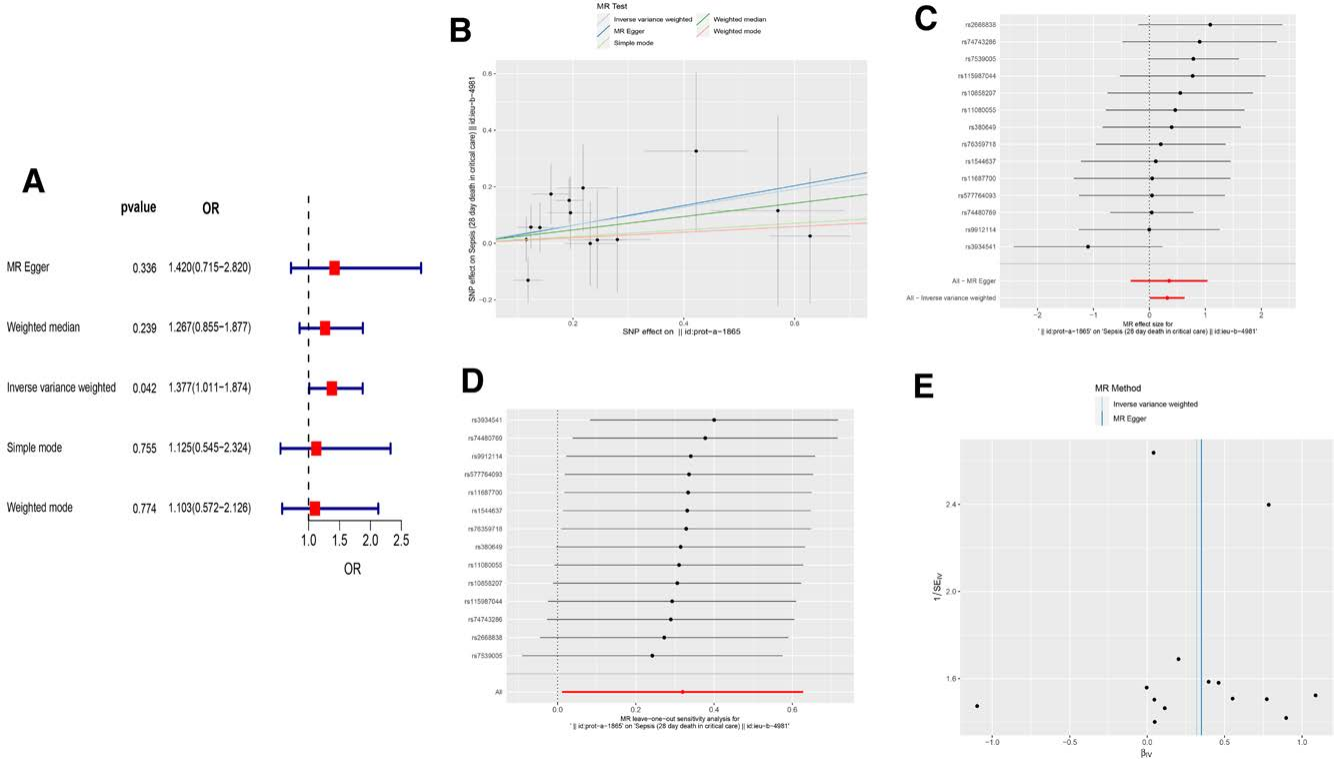
Mendelian randomization study results. (A) Forest plot showed the causal associations between MCEMP1 and sepsis by using different methods. (B) Scatter plot showing the causal effect of MCEMP1 on the risk of sepsis. (C) Forest plot showing the causal effect of each SNP on the risk of sepsis. (D) Leave-one-out plot to visualize causal effect of MCEMP1 on sepsis risk when leaving 1 SNP out. (E) Funnel plots to visualize overall heterogeneity of MR estimates for the effect of MCEMP1 on sepsis. MR = Mendelian randomization, SNPs = single nucleotide polymorphisms.

We used MCEMP1 as the exposure factor and sepsis as the outcome, incorporating 14 SNPs as instrumental variables. By conducting an MR analysis with these SNPs and applying the IVW method, we found a statistically significant association (OR = 1.376, 95% CI = 1.011–1.873, *P* = .042), suggesting a positive causal link between MCEMP1 and sepsis risk (Fig. 8A). However, when using 4 other methods, the results yielded *P* values exceeding 0.05, indicating a lack of significant associations. We further assessed the causal effects of each genetic variation on sepsis, as depicted in Figure 8B and C. Additionally, employing the leave-one-out analysis method to exclude each SNP individually demonstrated that no single SNP significantly altered the causal relationship between MCEMP1 and sepsis risk (Fig. 8D), confirming the robustness of our findings. The symmetry in the funnel plot for genetic variation (Fig. 8E) suggests the absence of heterogeneity among the factors considered. Finally, the results of the MR-Egger regression method, showing an intercept of −0.006 with a *P* value of .922, indicate no evidence of horizontal pleiotropy in our analysis.

## 4. Discussion

Sepsis has a high incidence rate, with 18 million people suffering from sepsis every year worldwide, and sepsis has a high mortality rate and is the leading cause of death in intensive care units.^[24]^ Despite increasing research on sepsis in recent years, sepsis mortality remains high, which may be due to the lack of effective biomarkers for the early diagnosis of sepsis. With the rapid development of gene chip technology, scholars began to explore the molecular mechanism of diseases from the genetic level. By quantifying the expression levels of myriad genes in biological specimens, which yielded genome-wide expression profiles, this study facilitated an investigation into the intricate regulatory interconnections among genes.^[25]^ This approach also enabled the screening of potential biomarkers crucial for early sepsis detection, diagnosis, and therapeutic intervention. In this study, bioinformatics technology was used to perform differential gene analysis on the data chip, and WGCNA was used to obtain the genes in the most sepsis-related modules. Eighty characteristic hub genes most closely related to sepsis were obtained. Through GO analysis, it was found that these hub genes were mainly involved in biological processes such as “defense response to bacteria,” “specific particles,” and “immune receptor activity.” Meanwhile, the KEGG pathway analysis mainly concentrates on the “NOD-like receptor signaling pathway,” “IL-17 signaling pathway,” and “C-type lectin receptor signaling pathway,” indicating active involvement in immune and inflammation-related processes. These findings suggest a potentially significant role of these genes in the development and progression of sepsis.

We employed machine learning methods (LASSO, RF, and SVM-RFE) to select 3 diagnostic genes (CD177, LDHA, and MCEMP1) to investigate potential sepsis biomarkers. Following this selection, we assessed the diagnostic efficiency of these core genes, revealing consistently high diagnostic effectiveness. Cluster of Differentiation 177 (CD177), a protein expressed on neutrophils, is encoded by a gene located on human chromosome 19, resulting in a membrane-bound protein.^[26]^ This protein is primarily present on the cell membrane of neutrophils and plays a role in the normal functioning of the immune system.^[27]^ In a murine sepsis model, Rasooli et al found that the expression of CD177 in the model was higher than in the control group.^[28]^ Research by Demaret et al showed that the expression of CD177 in neutrophils of patients with septic shock was significantly higher than in the control group.^[29]^ Studies have indicated that the protein corresponding to the CD177 gene can mediate the targeting of nanoparticles to the C5a receptor on neutrophils in humans and mice, and the C5a receptor is involved in neutrophil-mediated tissue damage associated with sepsis.^[30]^ Lactate dehydrogenase (LDH) is a rate-limiting enzyme that catalyzes the conversion of pyruvate to lactate in the last step of glycolysis.^[31]^ Because LDH exists only in the cytoplasm of tissue cells, its level in serum is usually low. Sepsis patients are attacked by bacteria or other pathogens, resulting in dysregulation of the systemic inflammatory response, destruction of tissue cell integrity, and LDH release into the blood, significantly increasing serum levels.^[32]^ LDH not only reflects the body inflammatory state but also has the value of monitoring the development of sepsis in microcirculation metabolism to a certain extent.^[33]^ Mast cell-expressed membrane protein 1 (MCEMP1) is involved in the regulation of mast cell differentiation or innate immune responses. Some scholars have established a mouse model of sepsis induced by cecal ligation and puncture to determine the expression of MCEMP1 and observed that MCEMP1 is highly expressed in septic mice.^[34]^ Loss of MCEMP1 can promote the activity of T lymphocytes and NK cells, increase the expression of immunoglobulin, inhibit the release of inflammatory cells, and reduce the apoptosis of T lymphocytes.^[34]^ In our study, the same results were obtained, and these 3 diagnostic genes (CD177, LDHA1, and MCEMP1) were all highly expressed in sepsis patient samples. Their expression levels were further verified in a separate external dataset, which is expected to become a new biomarker and therapeutic target for sepsis.

Immune cells play a vital role in the dysregulated response to sepsis.^[35]^ Studies have shown that immune cells often show an overreaction state in the early stage of sepsis and gradually develop into immune tolerance or immunosuppression with the progress of the disease.^[36]^ The immune response is overactive in the early stage of sepsis, which can activate various immune cells and produce many pro-inflammatory cytokines and chemokines.^[37]^ During the transition of sepsis into its immunosuppressive phase, a spectrum of lymphocyte dysfunctions and heightened apoptosis may ensue. Prior research has established that augmented apoptosis in CD4+, CD8+, and Th17 lymphocytes and a reduction in NK cell and B cell counts correlate with adverse outcomes in sepsis, encompassing shock and mortality.^[38]^ Immunosuppression is an essential factor in the progression of sepsis to shock or death: congenital and adaptive immune system disorders and dysfunction cause the pathological process of sepsis-induced immunosuppression.^[39]^ Mechanisms of dysregulation of the innate immune system include dysregulation of neutrophil recruitment and migration, abnormal differentiation and regulation of macrophages, suppression of immune function of activated dendritic cells, natural killer cytotoxicity and impaired cytokine production, and overactivation of the complement system.^[40]^ The CIBERSORT algorithm was used to analyze and compare the abundance of immune cell infiltration between the sepsis and healthy groups. The results revealed the presence of some immune cells, including “naive B cells,” “CD4 memory-activated T cells,” “gamma delta T cells,” “activated NK cells,” “monocytes,” and “neutrophils,” with statistically significant differences observed between the 2 groups. CD177, LDHA, and MCEMP1 exhibited significant correlations with a diverse range of immune cells. Consequently, the analysis of the association between these diagnostic genes and the immune infiltration in sepsis patients may offer novel targets and strategies for mitigating the immune dysregulation response in sepsis.

This study represents the inaugural exploration of the causal association between MCEMP1 levels and the risk of sepsis, employing 2-sample MR analysis. MR studies suggest that elevated serum MCEMP1 levels may increase the risk of sepsis and that there is a causal relationship. MR bears a conceptual resemblance to prospective randomized controlled trials (RCTs). However, it mitigates systemic biases that typically affect the outcomes of traditional observational studies, including confounding variables and reverse causation.^[41]^ The analysis was confined to participants of European descent to ascertain that the selected SNPs were devoid of any confounding associations between MCEMP1 and sepsis. Additionally, MR-Egger regression tests were conducted to validate our findings’ robustness, which revealed no indications of heterogeneity bias or directional horizontal pleiotropy. Notwithstanding these noteworthy results, the study is subject to certain limitations. Firstly, all data is collected from different datasets. Although data homogenization was performed in advance, bias between these data could not be eliminated. Secondly, the results obtained through bioinformatics analysis need to be verified by basic experiments in the future. Thirdly, MR analysis is based on a European database, so conclusions cannot be extended to other ethnic groups, limiting our results’ generality. Lastly, MR analysis of CD177 and LDHA was not performed due to the lack of SNPs in the GWAS database.

## 5. Conclusion

In summary, CD177, LDHA and MCEMP1 were identified as diagnostic biomarkers for sepsis. MCEMP1, in particular, demonstrated a positive causal relationship with sepsis risk. This study may increase the understanding of the potential molecular mechanism of sepsis and provide a new idea and entry point for further exploration of the development of novel diagnostic biomarkers and the treatment of sepsis.

## Author contributions

**Investigation:** Lin Li.

**Writing – original draft:** Zhongbo Xu.

**Writing – review & editing:** Zhongbo Xu.
